# Single-Cell RNA-Sequencing Reveals Peripheral T Helper Cells Promoting the Development of IgG4-Related Disease by Enhancing B Cell Activation and Differentiation

**DOI:** 10.3390/ijms241813735

**Published:** 2023-09-06

**Authors:** Zongfei Ji, Weiqi Lu, Sifan Wu, Yong Zhang, Dan Meng, Xiao Zhang, Xiaojuan Dai, Huiyong Chen, Lili Ma, Ying Sun, Lindi Jiang, Xiufang Kong

**Affiliations:** 1 Department of Rheumatology, Zhongshan Hospital, Fudan University, 180 Fenglin Road, Shanghai 200032, China; ji.zongfei@zs-hospital.sh.cn (Z.J.);; 2 Department of General Surgery, Zhongshan Hospital, Fudan University, 180 Fenglin Road, Shanghai 200032, China; 3 Department of Physiology and Pathophysiology, School of Basic Medical Sciences, Fudan University, Shanghai 200032, China; 4 Evidence-Based Medicine Center, Fudan University, Shanghai 200032, China

**Keywords:** IgG4-related disease, single-cell RNA-sequencing, peripheral T helper cell, TIGIT

## Abstract

Abnormal B cell differentiation plays a critical role in IgG4-related disease (IgG4-RD), but the underlying mechanism remains largely unknown. We investigated the cell landscape from three IgG4-RD retroperitoneal tissues and three control tissues using single-cell RNA-sequencing. Critical cell type or markers were further validated in the peripheral blood from the patients with IgG4-RD and healthy controls via flow cytometry as well as in the IgG4-RD and control tissue via immunofluorescence staining. The increases in B cells, plasma cells, and CD4^+^ T cells were found in IgG4-RD retroperitoneal tissue. Importantly, among CD4^+^ T cells, an increase in CD4^+^CXCR5^−^PD1^hi^ peripheral T helper (Tph) cells with a high expression of IL-21 and TIGIT was discovered in IgG4-RD tissue, which was further validated in peripheral blood of the patients with IgG4-RD. The Tph cell and TIGIT^+^ Tph cell proportion were remarkably higher in active IgG4-RD patients and correlated with disease activity. Moreover, TIGIT^+^CD4^+^ cells were able to promote B cell differentiation via IL-21. Our study revealed that Tph cells are increased in IgG4-RD and probably play critical roles in B cell differentiation through TIGIT-IL-21 axis. Peripheral Tph cell and TIGIT^+^Tph cell are potential markers for IgG4-RD disease activity.

## 1. Introduction

IgG4-related disease (IgG4-RD) is a systemic, inflammatory, autoimmune disease characterized by organ swelling or masses and elevated serum level of IgG4 [1]. According to data from the United States and Japan, the prevalence of IgG4-RD was 5.3–6.2/100,000 [2,3]. The peak age of onset is 50–60 years, with a male predominance of 62.7–65.3% [4,5]. The clinical presentation of IgG4-RD is remarkably heterogeneous. IgG4-RD can involve single or multiple organs, including the retroperitoneum, pancreas, biliary duct, salivary gland, lacrimal gland, etc. [5]. Because of the enlargement of the involved organs, patients present oppression symptoms or organ dysfunction such as hydronephrosis, recurrent pancreatitis, jaundice, submandibular mass, and eyelid swelling, and even have life-threatening conditions such as renal failure, stenosis of bile duct, and liver cirrhosis [6].

The pathogenesis of IgG4-RD is rather complicated and poorly investigated [7,8,9]. Multiple cell types participate in the development of this disorder [9,10,11,12,13,14,15,16,17]. Among them, B cell is most important, which can differentiate into IgG4-secreting plasma cell [18]. B cell activation and differentiation is induced by multiple kinds of T cell types, including T helper 2 (Th2) and T follicular helper (Tfh) cells. This process was thought to be mediated via Th2 cytokines (interleukin-4 [IL-4], IL-5, and IL-13) or Tfh cell cytokines (IL-21) [13,15,19,20], respectively. Treg cells are also involved in the regulation of B cells and fibrosis, which mainly functioned through IL-10 and TGF-β [21]. Among them, Tfh cells (CD4^+^CXCR5^+^PD1^+^ T cells) was believed to play critical roles in B cell differentiation and their interactions occurred mainly in the germinal centers of secondary lymphoid organs [22].

In recent years, a new type of CD4^+^ T cells, CXCR5^−^PD1^hi^ T peripheral helper (Tph) cells were found, which could also regulate B cell functions. In contrast to Tfh, this process mainly occurred outside of the germinal centers [13,23]. Moreover, it has been reported that the percentage of CD4^+^CXCR5^−^PD1^+^ T cells were increased in the peripheral blood of IgG4-RD patients [24,25]. Since IgG4-RD is a systemic disease, which can not only involve tissues rich of germinal centers such as salivary gland but also retroperitoneal tissue with less lymphoid structure [26]. Thus, it was assumed that Tph cells might also participate in plasma cell differentiation in IgG4-RD. However, its specific role in the pathogenesis of IgG4-RD remains unknown. Therefore, the cell landscape in IgG4-RD tissue and the underlying mechanism of Tph in this disorder are worthy of further investigation.

IgG4-related retroperitoneal fibrosis (IgG4-RPF) is a representative subtype of IgG4-RD with retroperitoneum involvement, in which the mass and fibrotic tissue usually surround the abdominal and iliac aorta, or constrict the ureters, leading to hydronephrosis or post-renal failure [27]. In the current study, we performed Single-cell RNA-sequencing (scRNA-seq) analysis using retroperitoneal tissue and aimed to elucidate the cell landscape in IgG4-RD tissue and explore more mechanisms in IgG4-RD pathogenesis.

## 2. Results

### 2.1. Study Population

**Figure 1:**
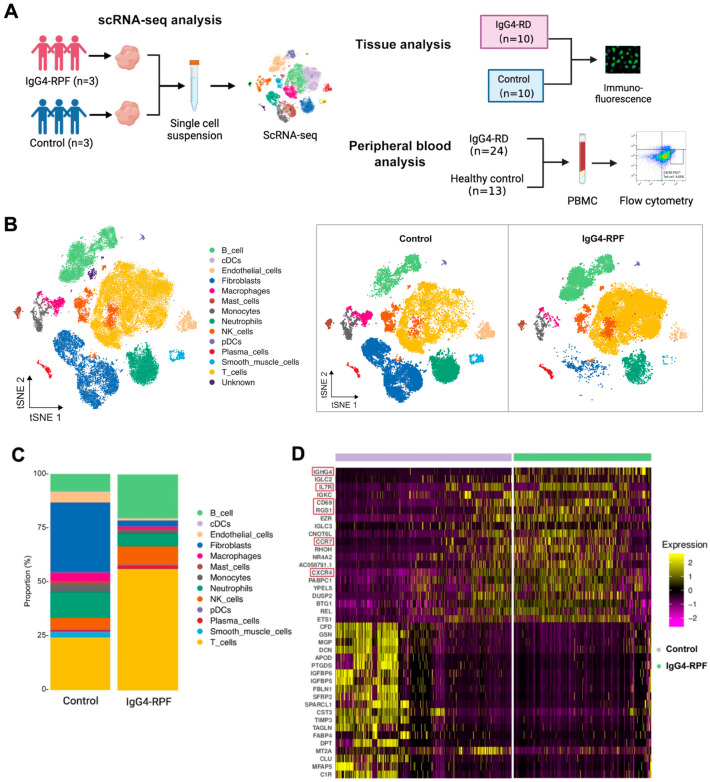
The study design and scRNA-seq profile of IgG4-RPF and control retroperitoneum. (**A**) Schematic diagram of the study design included the scRNA-seq analysis, tissue analysis, and peripheral blood analysis. (**B**) In total, 13 distinct cell types are identified in control and IgG4-RPF tissue. (**C**) The frequencies of different cell type in control and IgG4-RPF tissue are shown and the proportions of B and T cell are increased in IgG4-RPF group. (**D**) Heatmap showing the expression of IGHG4, IL7R, CD69, RGS1, CCR7, and CXCR4 were upregulated in the IgG4-RPF tissue than the control tissues. These genes are indicated in red boxes.

In this study, we not only used retroperitoneal tissues for scRNA-seq analysis but also recruited patients and control individuals for peripheral blood and tissue validation (Figure 1A). The demographic and clinical characteristics of the patients and control individuals are shown in Supplementary Table S1.

### 2.2. B and T Cell Proportions Are Increased in the Tissue of IgG4-RPF Patients

In this study, a total of 62,348 cells were obtained (44.5% from IgG4-RPF tissues and 55.5% from control retroperitoneum tissues). Based on their transcriptome, 13 distinct cell types were identified (Figure 1B) according to the cell markers genes (Supplementary Table S2), consisting of B cells, plasma cells, T cells, natural killer (NK) cells, monocytes, macrophages, neutrophils, classical dendritic cells (cDC), plasmacytoid DC (pDC), fibroblasts, mast cells, endothelial cells, and smooth muscle cells. Compared with those in control tissues, the proportions of B and T cell in IgG4-RPF tissue were increased (Figure 1C, Table S3). This result indicated that B cells and T cells might play important roles in the pathogenesis of IgG4-RPF. Thus, their subtypes were predominantly analyzed in subsequent analysis. Regarding to the whole transcriptome, multiple genes related with an inflammatory response and lymphocyte chemotaxis were increased in the IgG4-RPF group, including IGHG4, IL7R, CD69, RGS1, CCR7, and CXCR4 (Figure 1D). Among these DEGs, the high expression of IGHG4 (encoding the heavy chain of IgG4) confirmed this characteristic of IgG4-RD tissue. IL7R, CD69, and RGS1 are all important molecules involved in immune responses [28,29,30]. The increased expression of CCR7 and CXCR4 has not been reported in IgG4-RD before. According to the current research, CCR7 is mainly involved in lymph-node homing of naive and regulatory T cells via high endothelial venules [31]. This is also critical in IgG4-RD development. Regarding CXCR4, there was a study showing that its ligand CXCL12 was significantly increased in the serum and tissue of IgG4-RD patients and suggesting CXCL12/CXCR4 axis played important roles in the pathogenesis of this disorder [32].

### 2.3. The Active B Cells Are Increased in the Tissue of IgG4-RPF Patients

**Figure 2:**
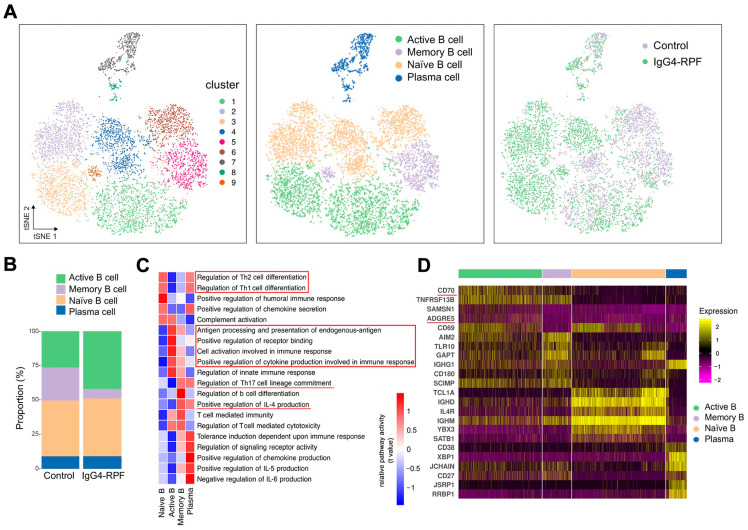
B cell and plasma cell analysis by scRNA-seq. (**A**) tSNE plots of B and plasma cell clusters, with cell types, and cells colored according to disease status in two different groups. (**B**) The cell subtypes of B cells and their fractions in the two groups. (**C**) GSVA of pathways in B cell subtypes. The important pathways related with immune response were indicated in red underlines or red boxes. (**D**) Heatmap showing marker genes of different subtypes of B cells. The costimulatory molecules CD70 and ADGRE5 (red underlines) are enriched in active B cells.

We re-clustered the B cells and plasma cells and identified nine clusters. Then, based on particular cell markers, we defined four subtypes (Figure 2A and Figure S1), consisting of active B cells (clusters 1 and 3), memory B cells (clusters 5 and 9), naïve B cells (clusters 2, 4 and 6), and plasma cells (clusters 7 and 8). Compared with the control group, the fraction of active B cells (characterized by CD69) was increased in the IgG4-RPF group, while the frequency of memory B cells was decreased (Figure 2B).

### 2.4. Gene Set Enrichment Analysis Reveals Enrichment of Cell Activation, Cytokine Secretion, and Receptor Binding Pathways in Active B Cells

According to the GSVA of different B cells subsets (Figure 2C), we found that the pathways related with Th1/Th2 cell differentiation were enhanced in both naïve B cells and plasma cells, while IL-4 production and Th17 cell differentiation were upregulated in memory B cells and plasma cells. In addition, chemokine secretion was increased in both naive B and plasma cells. The upregulation of receptor binding and antigen processing and presentation were observed in active B cells. The increased active B cells also actively participated in cell activation and cytokine secretion involved in immune response.

Specifically, the expression of several costimulatory molecules, including CD70 and ADGRE5 (CD97), were significantly increased in active B cells compared with those in other B cell subtypes (Figure 2D). These costimulatory molecules played important roles in T cell activation [33,34,35], indicating interactions of active B cells with T cells.

### 2.5. Tph Cells Are Increased in the Tissue of IgG4-RPF Patients

To investigate the role of T cells in the pathogenesis of IgG4-RD, T and NK cell profiles were further explored. They were clustered into nine sub-clusters and further defined as CD4^+^ T (cluster 1, 3, 4, 5), CD8^+^ T (cluster 2, 6), and NK (cluster 8–10) cells according to the specific cell markers (Figure S2A,B). Compared with the control tissues, the frequency of CD4^+^ T cells was increased, whereas the fractions of CD8^+^ T and NK cells were decreased (Figure S2C, Table S4).

**Figure 3:**
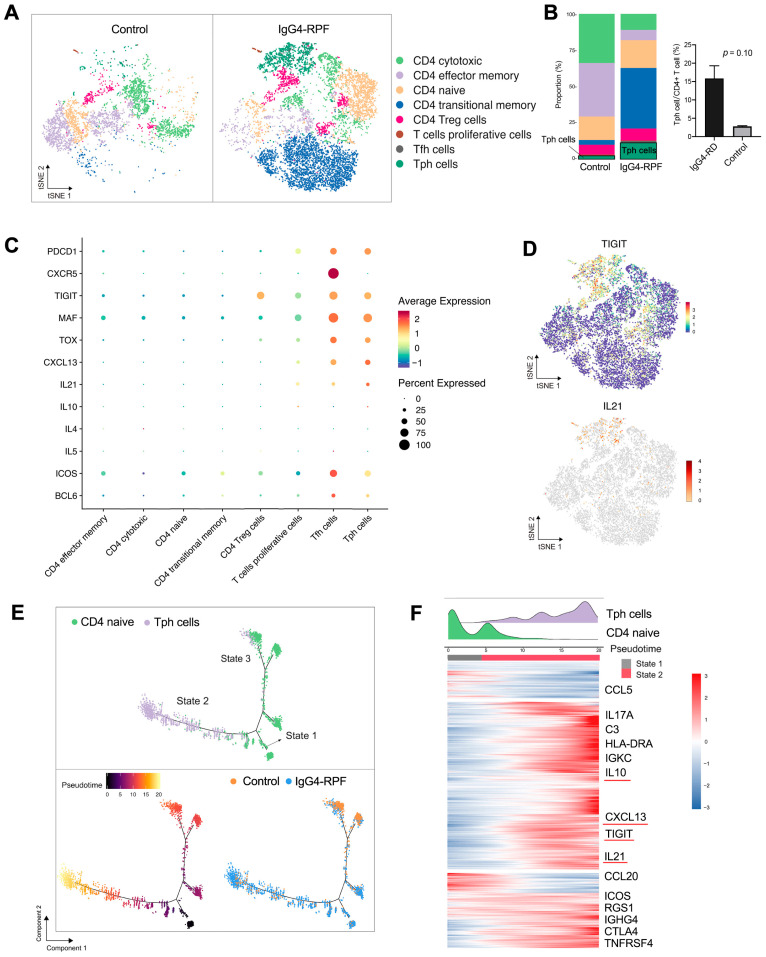
CD4^+^ T cell and Tph cell analysis by scRNA-seq. (**A**) t-SNE plot showing CD4^+^ T cell subtypes. (**B**) The fractions of different CD4^+^ T cell subtypes in the two groups. (**C**) scRNA data showing upregulation of PDCD1, CXCR5, TIGIT, MAF, TOX, CXCL13, and IL-21 in Tph cells. (**D**) t-SNE plot showing the expression of TIGIT and IL21 in CD4^+^ T cells. (**E**) Trajectory analysis of the transition states of naïve CD4^+^ T cell and Tph cells. (**F**) The highly variable genes expressed during the trajectory from naïve CD4^+^ cells to Tph cells. The distribution of cell types during the transition, along with the pseudotime, is shown at the top of the figure.

Additionally, CD4^+^ T cells were re-clustered into 14 clusters and identified eight subtypes (Figure 3A,B) according to their marker gene expressions (Figure S3A) [20,22,36,37,38,39], including CD4 cytotoxic T, CD4 effector memory T, CD4 naïve T, CD4 transitional memory T, CD4 Treg, proliferative CD4 T, Tfh, and Tph cells.

Among these subtypes, the fraction of Tph cells was significantly increased in the IgG4-RPF tissues (Figure 3B). This subset of cells expressed high levels of PDCD1 (PD1), immune regulator TIGIT, the transcription factors MAF and TOX, CXCL13, and B cell promoting factor IL-21, with a low expression of CXCR5 (Figure 3C,D). In addition, according to the GSVA, pathways including IL-10 production, the activation of an immune response, and T cell receptor signaling were upregulated in Tph cells (Figure S3B). Consistently, the high expression of IL-10 has also been reported in Tph cells in patients with systemic lupus erythematosus (SLE) [40,41].

To elucidate the lineage development of this special subset, trajectory analysis was performed, which indicated a transition from naïve CD4^+^ cells (State 1) to Tph cells (State 2) with increased pseudotime (Figure 3E). Compared with the naïve CD4^+^ T cells, multiple genes including TIGIT, CXCL13, IL-21, and IL-10 were significantly upregulated (Figure 3F), which was in accordance with the highly expressed genes of Tph cells shown in Figure 3C.

### 2.6. Tph Cells Were Increased in Both the Retroperitoneum Tissues and Salivary Glands in IgG4-RD Patients

**Figure 4:**
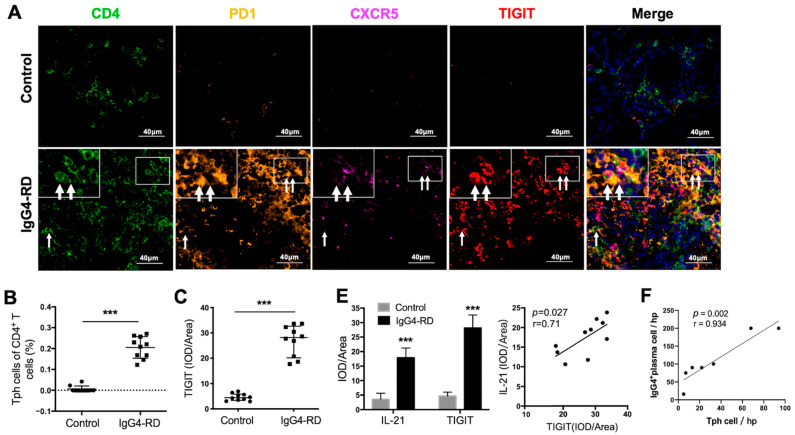

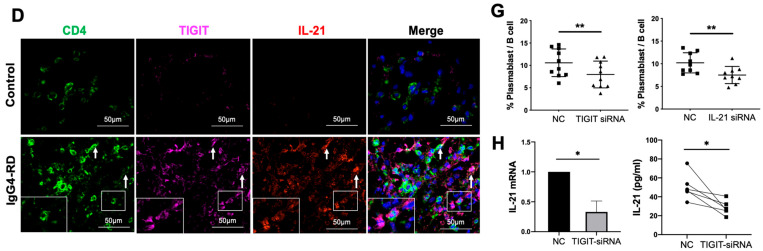
Tph cells in IgG4-RD tissues and positive regulation of Tph marker TIGIT on IL-21 expression. (**A**) Immunofluorescence images showing CD4^+^CXCR5^−^PD1^+^ Tph cell and its TIGIT expression in the control and IgG4-RD salivary gland tissues. The arrows showed the CD4^+^CXCR5^−^PD1^+^ Tph cells. The larger white boxes in the figure exhibited the magnification of the smaller ones. (**B**,**C**) Percentage of Tph cells among CD4^+^ T cells (**B**) and semi-quantitative analysis of TIGIT expression (**C**) on Tph cells determined by immunofluorescence staining (*n* = 10). (**D**) Co-expression of TIGIT and IL-21 in CD4^+^ T cells in IgG4-RD tissues. The arrows showed the CD4^+^TIGIT^+^IL-21^+^ cells. The larger white boxes in the figure exhibited the magnification of the smaller ones, with another CD4^+^TIGIT^+^IL-21^+^ cell inside. (**E**) Semi-quantitative analysis of TIGIT and IL-21 in the tissues of two groups and their correlation analysis (*n* = 10). (**F**) The number of IgG4^+^ plasma cells/HPF was positively correlated with that of Tph cells in the tissues of IgG4-RD. (**G**) Plasmablast detection in cocultures of naïve B cells and CD4^+^ T cells with TIGIT siRNA, IL-21 siRNA, or control scramble siRNA intervention. (**H**) Compared with the control scramble siRNA group, the expression of IL-21 significantly decreased in mRNA and protein levels in CD4^+^ T cells with TIGIT knockdown. *** *p* < 0.001, ** *p* < 0.01, * *p* < 0.05.

We further validated the existence of Tph cells in the tissue of IgG4-RD patients. Compared with the controls, the frequency of CD4^+^CXCR5^−^PD1^+^Tph cells was increased not only in the retroperitoneum tissues but also in the salivary glands in IgG4-RD patients (Figure 4A,B and Figure S4A). In addition, the high expression of TIGIT, an important immune regulatory molecule, was also confirmed in Tph cells (Figure 4A,C). Given that TIGIT has been reported to regulate cytokine production by CD4^+^ T cells [42] and IL-21 is an important cytokine that promotes plasma cell production [43], their co-expression was further explored in IgG4-RD tissue. The results showed that TIGIT and IL-21 were closely expressed in CD4^+^ cells in IgG4-RD tissue and their expression levels were positively correlated (Figure 4D,E). Moreover, Tph cells were positively correlated with IgG4^+^ plasma cells in the lesion tissue of IgG4-RD (Figure 4F).

### 2.7. Tph Cells Promote B Cell Differentiation by TIGIT-IL-21 Axis In Vitro

To examine the TIGIT-IL-21 axis in Tph cell-mediated B cell differentiation, CD4^+^ cells from patients with IgG4-RD were co-cultured with naïve B cells. Importantly, the differentiation of naïve B cells into plasmablasts was decreased when TIGIT or IL-21 in CD4^+^ T cells was knocked-down (Figure 4G and Figure S4B,C). To clarify the role of TIGIT in IL-21 secretion, TIGIT was knocked-down in CD4^+^ T cells and it showed that the expression of IL-21 was significantly decreased in both mRNA and protein levels (Figure 4H). However, the expression of other cytokines that potentially regulate B cell functions such as IL-4, IL-5, and IL-10 were not affected (Figure S4D). These results strongly suggested that the increased Tph cells in IgG4-RD might promote B cell differentiation via TIGIT-IL-21 axis.

### 2.8. Peripheral Tph Cells and TIGIT^+^ Tph Cells Are Positively Correlated with IgG4-RD Activity

**Figure 5:**
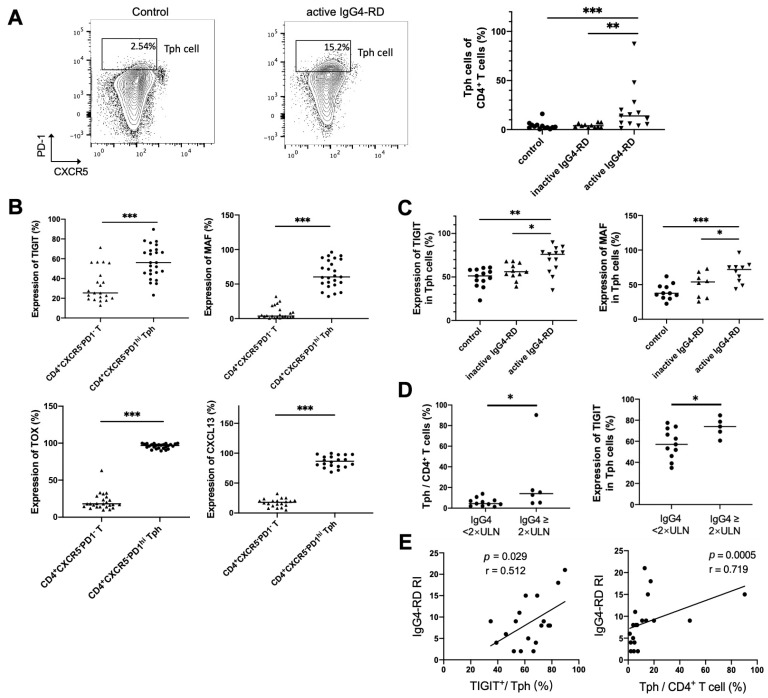
Tph cells and its marker expression in the peripheral blood and their relationships with clinical indicators. (**A**) Peripheral CD4^+^CXCR5^−^PD1^hi^ Tph cells significantly increased in patients with active IgG4-RD compared with the controls. (**B**) Higher expressions of TIGIT and MAF in CD4^+^CXCR5^−^PD1^hi^ Tph cells than those in CD4^+^CXCR5^−^PD1^−^ T cell. (**C**) Higher levels of TIGIT, MAF, TOX, and CXCL13 in Tph cells from patients with active IgG4-RD than those from inactive patients or healthy controls. (**D**) A higher percentage of Tph cells among CD4^+^ T cells and higher expressions of TIGIT in Tph cells in patients with raised serum IgG4 levels. (**E**) TIGIT expression in Tph cells and the percentage of Tph cells among the CD4^+^ T cells correlated positively with IgG4-RD responder index (RI). * *p* < 0.05, ** *p* < 0.01, *** *p* < 0.001. Data are represented as median.

To clarify the relationship between Tph cell and disease activity, Tph cells were also detected in the peripheral blood of patients with IgG4-RD. It showed that the frequency of CD4^+^CXCR5^−^PD1^hi^ Tph cells was significantly increased in active IgG4-RD patients (Figure 5A). Expressions of other enriched genes shown in scRNA-seq data, including TIGIT, CXCL13, and the transcription factors MAF and TOX, were also detected in peripheral Tph cells. This showed that these markers were remarkably increased in CD4^+^CXCR5^−^PD1^hi^ Tph cells than those in CD4^+^CXCR5^−^PD1^−^ T cells (Figure 5B). Furthermore, we found that the levels of MAF and TIGIT on Tph cell were significantly higher in active patients than those in inactive patients or healthy controls (Figure 5C). Importantly, the proportions of Tph/CD4^+^T and TIGIT^+^/Tph were higher in the patients with high serum IgG4 levels and were positively correlated with IgG4-RD responder index (RI) (Figure 5D,E).

### 2.9. Tph Cell and B Cell Interactions Are Upregulated in IgG4-RPF

**Figure 6:**
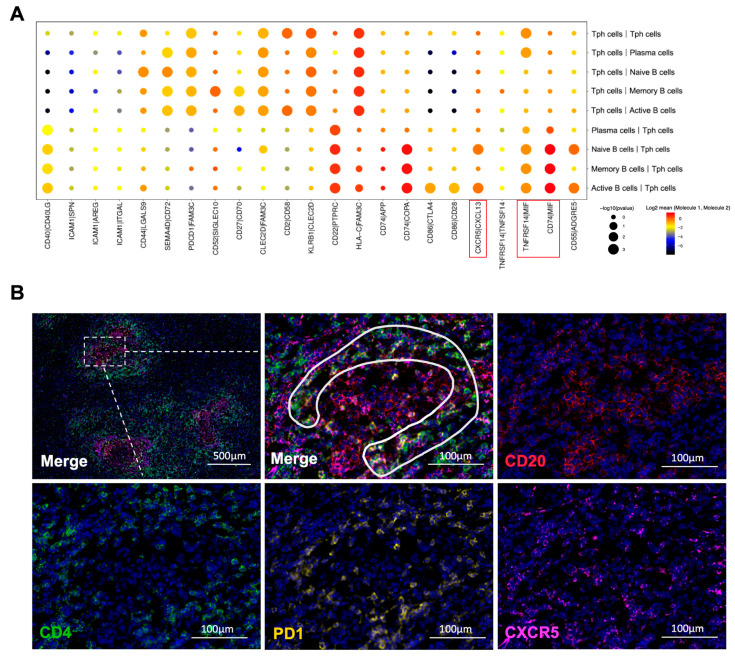
Interactions between B cells and CD4^+^ T cells. (**A**) Receptor–ligand pairs between Tph cells and B cell subtypes in the IgG4-RPF group. The interactions of CXCR5/CXCL13, TNFRSF14/MIF, and CD74/MIF (red boxes) were enhanced between different B cell subtypes and Tph cells. (**B**) The distribution of CD4^+^CXCR5^−^PD1^+^ Tph cell (inside the white line) and CD20^+^ B cells in a lymphoid follicles-like structure in IgG4-RD tissues.

Based on the scRNA-seq results, we further analyzed the cell communication between B cell subtypes and CD4^+^ T cell subtypes, especially Tph cells. The cell interactions (Figure S5A) showed that Tph cells engaged in strong cross-talk with naïve B, active B, memory B, and plasma cells (Figure 6A and Figure S5B). Meanwhile, in the IgG4-RD tissue, a close spatial localization of Tph cells and B cells was also confirmed using immunofluorescence staining. As shown in Figure 6B, Tph cells were surrounding the B cells in a lymphoid follicles-like structure.

Furthermore, according to the interaction analysis (Figure 6A), we found that the CXCR5/CXCL13 interaction was upregulated between active B cells or naïve B cells and Tph cells, which was also consistent with the high expression of CXCL13 in Tph cells (Figure 3C and Figure 5B). This result strongly indicated a chemotactic effect of Tph on these two subtypes of B cells. In addition, TNFRSF14/MIF and CD74/MIF interactions were also increased between B cell subtypes and Tph cells (Figure 6A). MIF can mediate the survival, proliferation, and migration of B cells [44], indicating that Tph located in IgG4-RPF lesion tissue possess a strong ability to recruit different subtypes of B cells.

## 3. Discussion

IgG4-RD is an immune-mediated fibro-inflammatory disease involved multiple organs. Its pathogenesis was largely unexplored due to the low incidence. By dissecting the cell landscape of the retroperitoneum tissue from IgG4-RD patients, this study discovered a remarkable increase in active B cells and CD4^+^ T cells. Multiple processes involved in immune response were enhanced in different B/plasma cell subsets. Importantly, Tph cell, a subset of CD4^+^ T cells, signatured with high expression of IL-21 and TIGIT was found to be related with disease activity and probably promoted B cell differentiation via TIGIT/IL-21 axis.

Recently, several studies have performed scRNA-seq in IgG4-RD patients. One study investigated the CD4^+^ T cells in the salivary glands of IgG4-RD patients and Kimura disease patients [20]. They placed emphasis on Tfh cells rather than Tph. In addition, CD4^+^ T cells with a high expression of NKG7, GZMA, GZMK have been observed, which might correspond to the CD4 cytotoxic cell type in our results. Another study from Wu X. et al. performed scRNA-seq using the PBMCs from IgG4-RD patients and healthy controls [45]. They focused on the increased CD14^+^ monocytes, CD8 central memory T, and CD8 cytotoxic T cells, which was also distinguished from our study. Recently, there was another scRNA-seq study investigating the landscape of T-B cell interactions in IgG4-RD tissue, which found Th1 and Th2-related inflammatory responses in IgG4-RD involved tissue [46]. Thus, different sample types and disease stages may contribute to the discrepancies in these findings.

In this study, B cells and T cells were specifically analyzed due to their pathogenic roles in IgG4-RD. The infiltration of B cells and plasma cells is a characteristic pathological manifestation of IgG4-RD [47]. In the present study, an increase in active B cells was found among different B cell subtypes. This subset highly expressed multiple costimulatory molecules, such as CD70 and CD97, which indicated that active B cell played a major role in the pathogenesis of IgG4-RD.

Through GSVA, it demonstrated that genes involving Th1/Th2 and Th17 cell differentiation were enriched in different subsets of B cells, implying an important role of B cell in T cell differentiation and activation. It has been known that Th2 and Tfh cells were increased in IgG4-RD and played important roles in B cell differentiation and IgG4 production [19,20,48,49]. Recently, an increase in Th17 was also found in this disorder [50], indicating multiple T subsets were activated in IgG4-RD. Consistently, Shaozhe Cai [46] reported a landscape of T and B lymphocytes interaction and synergistic effects of Th1 and Th2 type response in IgG4-RD tissue. A cross talk between B cell and T cell has also been put forward by Touzani F. et al. in IgG4-RD in 2019 [51]. Thus, B cells and T cells probably closely interacted in the pathogenesis of IgG4-RD. Moreover, positive regulations of different cytokines, chemokines, or receptor binding in different B cell subsets also supported their roles in cell interactions. For example, the upregulation of IL-4 in memory B cells implied its role in Th2 differentiation [19].

Through analyzing clusters of CD4^+^ T cells, the proportion of Tph cells was found to be significantly increased in IgG4-RPF tissue. Tph cell is a novel CD4^+^ T cell cluster and was first found in RA [22]. The increased Tph cell has also been reported in other autoimmune disease such as SLE, Sjogren’s syndrome, and multiple sclerosis [52,53,54]. Recent studies found that this type of cells can also regulate B cells functions [22,23,55]. Tph cells share several features with Tfh cell, including the high expression of IL-21, CXCL13, MAF, PD-1, and ICOS. According to current studies, both Tfh and Tph contributed pathogenic B cell activation [56,57]. However, it was reported that the regulation of Tph on B cells mainly happened outside of the germinal centers, which is different from Tfh cells [22,23]. Moreover, according to the scRNA-seq data in the present study, the percentage of Tph cells was far greater than that of Tfh cells in the IgG4-RD group, indicating an important role of Tph cells in the lesion tissues with less germinal center formation, such as retroperitoneum.

It has been reported that the frequency of Tph cells were increased in the peripheral blood of IgG4-RD patients and correlated with serum IgG4 and peripheral plasmablast level [24,25]. However, the existence of Tph cells in the lesion tissue of IgG4-RD and its role in the pathogenesis of this disorder is still unclear. In the current study, Tph cells and their markers were also verified in different tissue as well as peripheral blood in patients with IgG4-RD. The positive correlations between peripheral Tph cells and disease activity, IgG4-RD RI, and IgG4 levels further confirmed their important role in the pathogenesis of IgG4-RD.

Tph was characterized by highly expression of multiple cell markers, including CXCL13, TIGIT, and MAF, etc. CXCL13 was a ligand of CXCR5, which was also shown in the interaction analysis between Tph and B cells, indicating the regulation of Tph on B cell function. This was consistent with the phenomenon in RA [58]. In the present study, the expression of TIGIT on Tph cells was closely correlated with disease activity in patients with IgG4-RD. It is an immune receptor-mediated signaling pathway in cytokine expression, such as IL-21 [59], which is an important cytokine in B cell differentiation and IgG4 production [60]. By immunofluorescence staining, co-expression of TIGIT and IL-21 was also observed in CD4^+^ T cells in IgG4-RD tissue. Furthermore, the regulatory effect of TIGIT on IL-21 expression was confirmed in our in vitro study. These findings implicated that TIGIT might be a critical factor in mediating Tph and B cell interaction.

In this study, para-tumor or para-inflamed tissue were used in scRNA-seq as control samples and the sample size was relatively small; thus, a further verification with larger sample sizes needs to be performed. In addition, the role of Tph remains to be expanded in other organ tissues involved in IgG4-RD to confirm its pathogenic mechanism in B cell differentiation. Moreover, our study was based on the scRNA-seq analysis using a cross-sectional design. Thus, the results need to be validated through additional experiments and longitudinal studies in the future.

In conclusion, through scRNA-seq profiling of IgG4-RPF tissue, different B/plasma cells probably have multiple unexplored functions in the pathogenesis of IgG4-RD, especially active B cells. Tph cells probably play a critical role in B cell differentiation via TIGIT-IL-21 axis. Peripheral Tph percentage and its TIGIT levels are potential novel biomarkers for disease activity in IgG4-RD. These findings provide a novel pathogenic mechanism for IgG4-RD and will facilitate the development of effective therapeutics in future studies.

## 4. Materials and Methods

### 4.1. Patients

In total, 39 IgG4-RD patients and 24 control individuals were included in this study. Patients were recruited mainly from the Department of Rheumatology, Zhongshan Hospital, Fudan University between 1 January 2020 and 31 October 2021. Patients were diagnosed as definite IgG4-RD according to the 2011 Japanese Comprehensive Clinical Diagnostic Criteria [61] and those with retroperitoneum involvement were classified as IgG4-RPF. Patients with concurrent carcinoma, infection, hematologic disease, or other rheumatic disease were excluded. Two expert rheumatologists were responsible for the diagnosis and disease activity assessment. Active IgG4-RD was defined as new onset disease, or progressive disease with clinical symptoms or imaging findings after remission and with or without elevation of serum IgG4 level. Inactive IgG4-RD was defined as improved symptoms with a decline in IgG4-RD responder index (RI) [61] by ≥2 after at least 3 months of treatment [62,63].

A schematic diagram of the study design is shown in Figure 1A. In the discovery process, five untreated IgG4-RPF patients and one control patient with a benign retroperitoneal tumor were recruited, providing three IgG4-RPF retroperitoneal tissues and three control tissues (1 para-tumor tissue and 2 para-inflamed tissue) for scRNA-seq. In the validation process, 24 IgG4-RD patients (13 active, 11 inactive) and 13 healthy controls were enrolled for peripheral blood analysis. These healthy controls were recruited from subjects for routine medical checkups in our hospital during the same period. For tissue validation, we recruited 10 IgG4-RD patients (5 with salivary gland involvement, 5 with IgG4-RPF) and 10 control patients (5 Sjögren’s syndrome and 5 benign retroperitoneal tumor). These patients or controls supplied salivary gland lesion tissues (5 vs. 5), retroperitoneum lesion, or para-benign retroperitoneal tissues (5 vs. 5) for immunofluorescence staining.

This study was approved by the Ethics Committee in Zhongshan Hospital of Fudan University (B2020-260R) and conformed to the ethical guidelines of the 1975 Declaration of Helsinki. Written informed consent was obtained from all participants.

### 4.2. ScRNA-Seq

#### 4.2.1. Preparation of Single-Cell Suspensions

The single-cell isolation and sequencing were performed in Shanghai OE Biotech. Co. Ltd. (Shanghai, China). Samples from resection surgery were isolated and transported rapidly to the research facility. Each sample was minced on ice to less than 1 mm cubic pieces, and digested using collagenase type I (Gibco, Grand Island, NY, USA). Then, samples were centrifuged at 300 rcf for 30 s at room temperature and removed the supernatant without disturbing the cell pellet. Samples were washed with phosphate-buffered saline (PBS) containing 0.04% BSA (400 µg/mL) and then centrifugation was performed at 300 rcf for 5 min. The cell pellet was resuspended in red blood cell lysis buffer and incubated for 10 min at 4 °C and then resuspended in 1 mL PBS containing 0.04% BSA. Next, samples were filtered over Scienceware Flowmi 40 µm cell strainers (VWR). After tissue dissociation, cell concentration and cell viability were determined using the Trypan Blue staining method to assess the quality of the samples.

#### 4.2.2. Data Acquisition and Quality Control of the Single Cells

The 10× Genomics Cell Ranger software pipeline (version 5.0.0) was used to demultiplex cellular barcodes. Reads were mapped to the genome and transcriptome using the STAR aligner. Down-sample reads were required to generate normalized aggregate data across samples and produce a matrix of gene counts versus cells. We processed the unique molecular identifier (UMI) count matrix using the R package in Seurat (version 3.1.1) [64]. To remove low-quality cells and likely multiplet captures, we filtered out cells with UMI/gene numbers out of normal range for each cell. Low-quality cells where >10% of the counts belonged to mitochondrial genes were also discarded. Additionally, we applied the DoubletFinder package (version 2.0.2) [65] to identify potential doublets. After applying these quality control criteria, the remaining single cells were included in downstream analyses. Full details of the methods used for quality control are shown in the Supplementary Methods section.

#### 4.2.3. Gene Analysis, Clustering, and Identification of the Single Cells

The most variable genes were selected using the FindVariableGenes function in Seurat [66]. Principal component analysis (PCA) was performed to reduce the dimensionality with the RunPCA function in Seurat [64]. Graph-based clustering was performed to cluster cells according to their gene expression profile by Seurat [64]. The FindAllMarkers function in Seurat was used to identify marker genes of each cluster. Multimodal intersection analysis (MIA) [67] was then used with the reference transcriptomic datasets [36,68,69,70] to identify cell types of each single cell. Differentially expressed genes (DEGs) were identified using the FindMarkers function in Seurat [64]. *p*-values < 0.05 and |log_2_fold change| > 0.58 was set as the threshold for significantly differential expression. Full details of the methods are shown in the Supplementary Methods section. Marker genes of certain cell subsets were also evaluated by gene diff value.

#### 4.2.4. Pseudotime Analysis

We determined the developmental pseudotime and selected ordering genes with the Monocle2 package (version 2.9.0) [71]. Dimensional reduction clustering analysis was performed with the reduceDimension function, followed by trajectory inference with the orderCells function using default parameters. Gene expression was plotted with the plot_genes_in_pseudotime function to track changes over pseudotime.

#### 4.2.5. Cell–Cell Communication Analysis and Gene Set Variation Analysis (GSVA)

Cell–cell communication was analyzed using CellPhoneDB to identify biologically relevant ligand-receptor (LR) interactions [72]. To define cell–cell communication networks, we linked any two cell types where the ligand was expressed in the former cell type and the receptor in the latter. R packages Igraph (version 1.2.4.1) and Circlize (version 0.4.8) were used to display the cell–cell communication networks. For GSVA, the GSEABase package (version 1.44.0) [73] was used to load the gene set file which was downloaded and processed from the KEGG database. Full details of the methods are shown in the Supplementary Methods section.

### 4.3. Flow Cytometry

The frequency of peripheral T helper (Tph) cells, a specific type of CD4^+^ T cell identified by scRNA-seq of the peripheral blood of patients with IgG4-RD, was determined by routine flow cytometry. The expression of TOX, TIGIT, CXCL13, and MAF in the CD4^+^CXCR5^−^PD1^hi^ Tph cells was also detected. Briefly, fresh peripheral blood samples were obtained from each subject and stained for specific surface markers using fluorochrome-labeled antibodies. After cell permeabilization using Fix/Perm buffer (BD Biosciences), intracellular markers (TOX, TIGIT, CXCL13, and MAF) and Tph markers (CD4, CXCR5, and PD1) were stained. The following antibodies were used: anti-CD4 (BV510, Biolegend, San Diego, CA, USA, 1:50), anti-CXCR5 (BB515, BD Biosciences, San Diego, CA, USA, 1:25), anti-PD1 (APC, BD Biosciences, 1:5), anti-TOX (PE, Miltenyi, Bergisch Gladbach, Germany, 1:50), anti-TIGIT (BV421, Biolegend, 1:25), anti-CXCL13 (PE, Thermo, Waltham, MA, USA, 1:10), and anti-MAF (EF 450, Thermo, 1:25). In the T/B cell co-cultures, CD19^+^CD27^+^CD38^+^ plasmablasts were detected. The following antibodies were used: anti-CD19 (APC-CY7, BD Biosciences, 1:20), anti-CD27 (PE, BD Biosciences, 1:5), anti-CD38 (PE-Cy7, BD Biosciences, 1:20). Flow cytometric data were acquired using a FACSAria III (BD Biosciences) and analyzed with FlowJo (version 10.4). The gating strategy of Tph cells is shown in Figure S6.

### 4.4. Immunohistochemistry and Immunofluorescence Staining

Immunohistochemistry (IHC) and multi-color immunofluorescence were performed to detect protein expression in tissues of IgG4-RD patients or controls. The rabbit monoclonal anti-IgG4 antibody (EP4420, Abcam, Cambridge, UK) was used for IHC staining to determine the IgG4^+^ plasma cell in the tissue. In the immunofluorescence staining, co-staining of CD4 (1:100, BX50023, Shanghai Recordbio, Shanghai, China), CXCR5 (1:1000, ab254415, Abcam), TIGIT (1:100, MAB78982, Novus, Centennial, CO, USA), PD-1 (1:200, 86163S, Cell Signaling Technology, Boston, MA, USA) was performed to detect the existence of TIGIT^+^ Tph cells. Co-staining of CD4, TIGIT, and IL-21 (1:200, 17625-1-AP, Proteintech, Chicago, IL, USA) was conducted to determine the co-expression of TIGIT and IL21 in the CD4^+^ T cell. Co-staining of CD20 (1:1000, 60271-1-Ig, Proteintech) and CD137L (1:500, ab64912, Abcam) was carried out to detect CD137L expression in B cells. This immunofluorescence staining was performed using a three- or four-color Fluorescence kit (Shanghai Recordbio Biological Technology Co. Ltd., Shanghai, China) based on the tyramide signal amplification (TSA) technology according to the manufacturer’s instructions. Immunostained sections were scanned by Panoramic Scan (MIDI II, 3D Histech Ltd., Budapest, Hungary). Semi-quantification of the protein expression was analyzed using Image-Pro Plus 6.0 and presented as the ratio of integrated optical density (IOD) to the relevant area. For each slide, the semi-quantification process was based on five viewing fields within the area of greatest inflammatory infiltration (400× magnification). The numbers of IgG4^+^ plasma cells and Tph cells were determined by manually counting the cells in five high-power fields (HPF) and calculating the average (400× magnification). The percent of Tph cells in CD4^+^ T cells was then calculated. One pathologist who was blinded to the disease information was responsible for the evaluation for all the specimens.

### 4.5. Isolation of CD4^+^ T and B Cells and Cell Culture

Ten milliliters of peripheral blood were obtained from each patient. PBMC was collected using Ficoll–Paque Gradient method, from which CD4^+^ T cells were further isolated using CD4 microbeads (Miltenyi Biotec, Bergisch Gladbach, Germany). At last, around 2 × 10^6^ cells were obtained. Then, the isolated CD4^+^ T cells were suspended in complete RPMI-1640 medium at a density of 10^6^/mL and seeded in 24-well plate with 1 mL each well. After 48 h, the RNA and protein expression of various cytokines in T cells were detected. For T/B cell co-cultures, isolated CD4^+^ T cells (1 × 10^5^) and autologous B cells (B cell isolation kit, Miltenyi Biotec) were incubated at a 1:1 ratio in 96-well in RPMI-1640 in the presence of LPS (5 μg/mL, Invitrogen, Carlsbad, CA, USA) and SEB (0.2 μg/mL, MCE, Monmouth Junction, NJ, USA). At day 7, cells were harvested and the frequency of plasmablasts detected by flow cytometry.

### 4.6. Transfection of Small Interfering RNA (siRNA)

Cells in 24-well plate were transfected with 0.5 μL siRNA with jetPRIMEtransfection reagent (Polyplus) according to the manufacturer’s instructions. After 48 h, cells were harvested for subsequent experiments. The siRNA sequences for human TIGIT (GGGAGTACTTCTGCATCTA), IL-21(GCGTCAACTTATAGATATT), and the control scramble siRNA were synthesized by Guangzhou RiboBio (Guangzhou, China).

### 4.7. Enzyme-Linked Immunosorbent Assay (ELISA)

Cell supernatant were collected from CD4^+^ T cell culture. IL-21 level was detected using human IL-21 ELISA kit (ab119542, Abcam) according to the instructions.

### 4.8. RNA Extraction and Real-Time Quantitative PCR

RNA was extracted from CD4^+^ T cell using Micro Cells Total RNA Extraction Kit (Simgen, Hangzhou, China) and quantified using spectro-photometry (Denovix DS-11). The PrimeScript RT reagent kit (RR047A, Takara Biotechnology, Dalian, China) and Hieff Direct Taq DNA Polymerase (YEASEN, Shanghai, China) were used for reverse transcription and later qualification. The primers used in this study are listed in the Supplementary Methods section. The comparative Ct method was used to calculate gene expression and β-actin was taken as an internal reference.

### 4.9. Statistical Analysis

The continuous variables were summarized as means ± standard error (SE) for the normally distributed data, while non-normally distributed data were represented by median and quartiles. The categorical variables were represented as frequencies and percentages. For comparisons between groups, Student’s *t*-test or a Mann–Whitney U-test were used as applicable. A Wilcoxon matched-pairs signed rank test was used for paired-group comparisons. Correlations between different parameters were analyzed using Spearman’s correlation analysis. A two-tailed *p* < 0.05 was considered statistically significant. Statistical analysis was performed using SPSS for Windows, Version 20.0 (IBM Corp., Armonk, NY, USA).

## Data Availability

The scRNA-seq data presented in this study are openly available in GEO database (GSE231920).

## Supplementary Materials

The supporting information can be downloaded at: https://www.mdpi.com/article/10.3390/ijms241813735/s1.
