# Supplementary material for: Single-Cell RNA-Sequencing Reveals Peripheral T Helper Cells Promoting the Development of IgG4-Related Disease by Enhancing B Cell Activation and Differentiation
Source: Int J Mol Sci. 2023 Sep 6;24(18):13735. doi: 10.3390/ijms241813735 (PMC10530310; doi:10.3390/ijms241813735)
Supplement: Supplementary file 1 [file ijms-24-13735-s001.zip › Supplementary Figures.pdf]

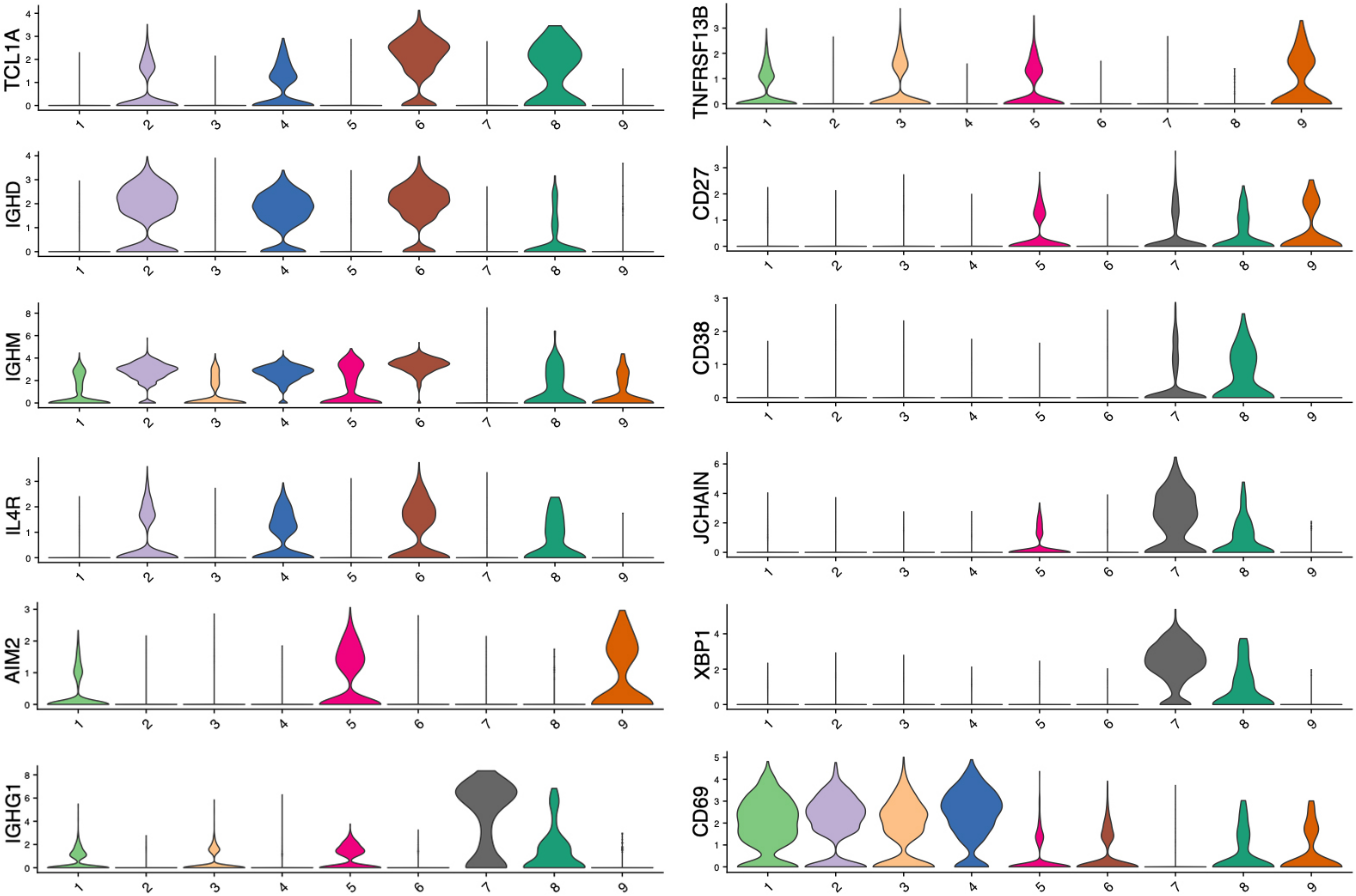

Supplementary Figure S1. Violin plots of selected marker genes in the different B cell clusters

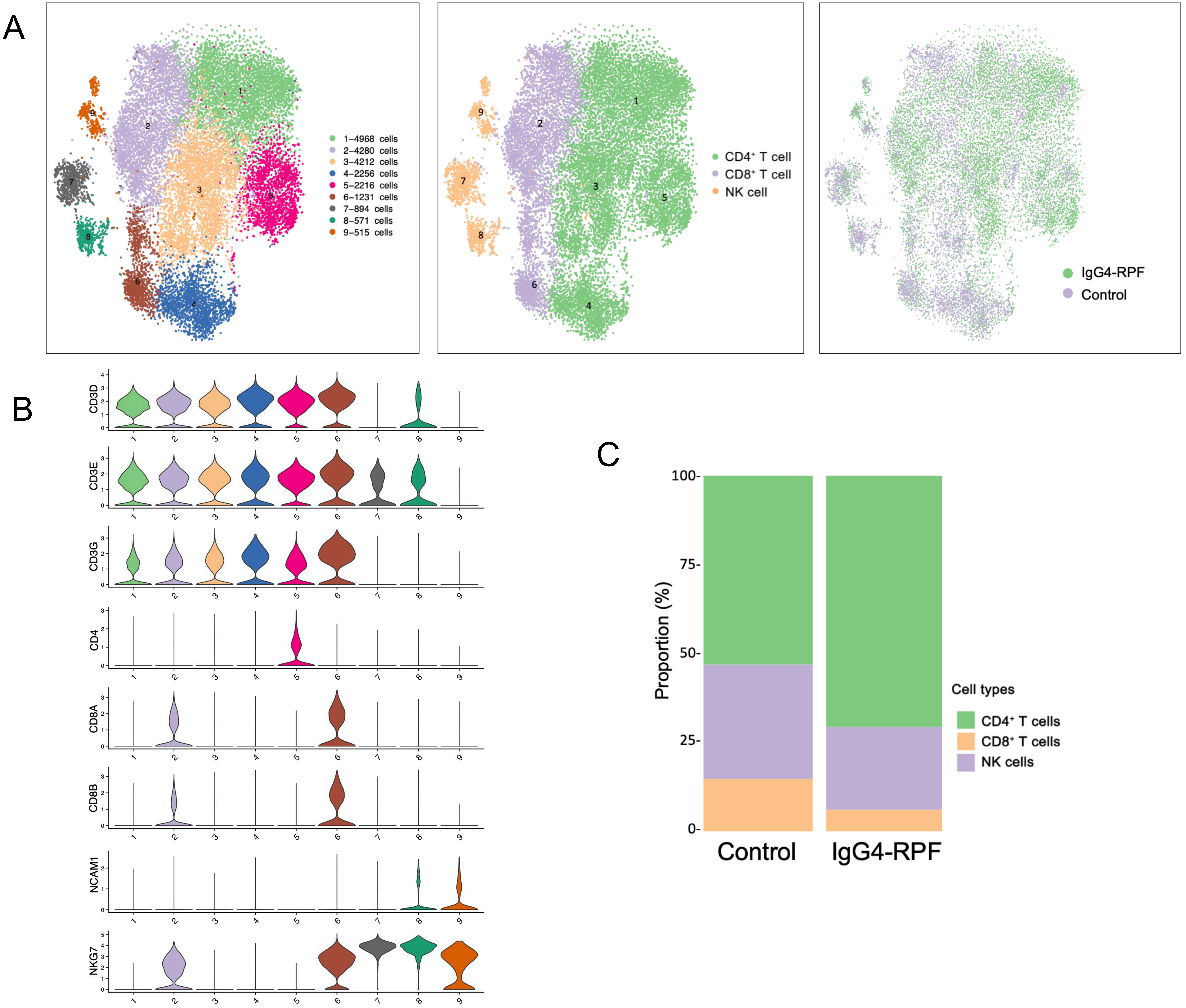

Supplementary Figure S2. T and NK cell analysis by scRNA-seq. (A) tSNE plots of T/NK cell clusters, with cell types, and cells colored according to disease status in the two different groups. **(B)** Marker gene expressions in the different T/NK cell clusters. **(C)** The proportion of different T/NK cell types in the two groups.

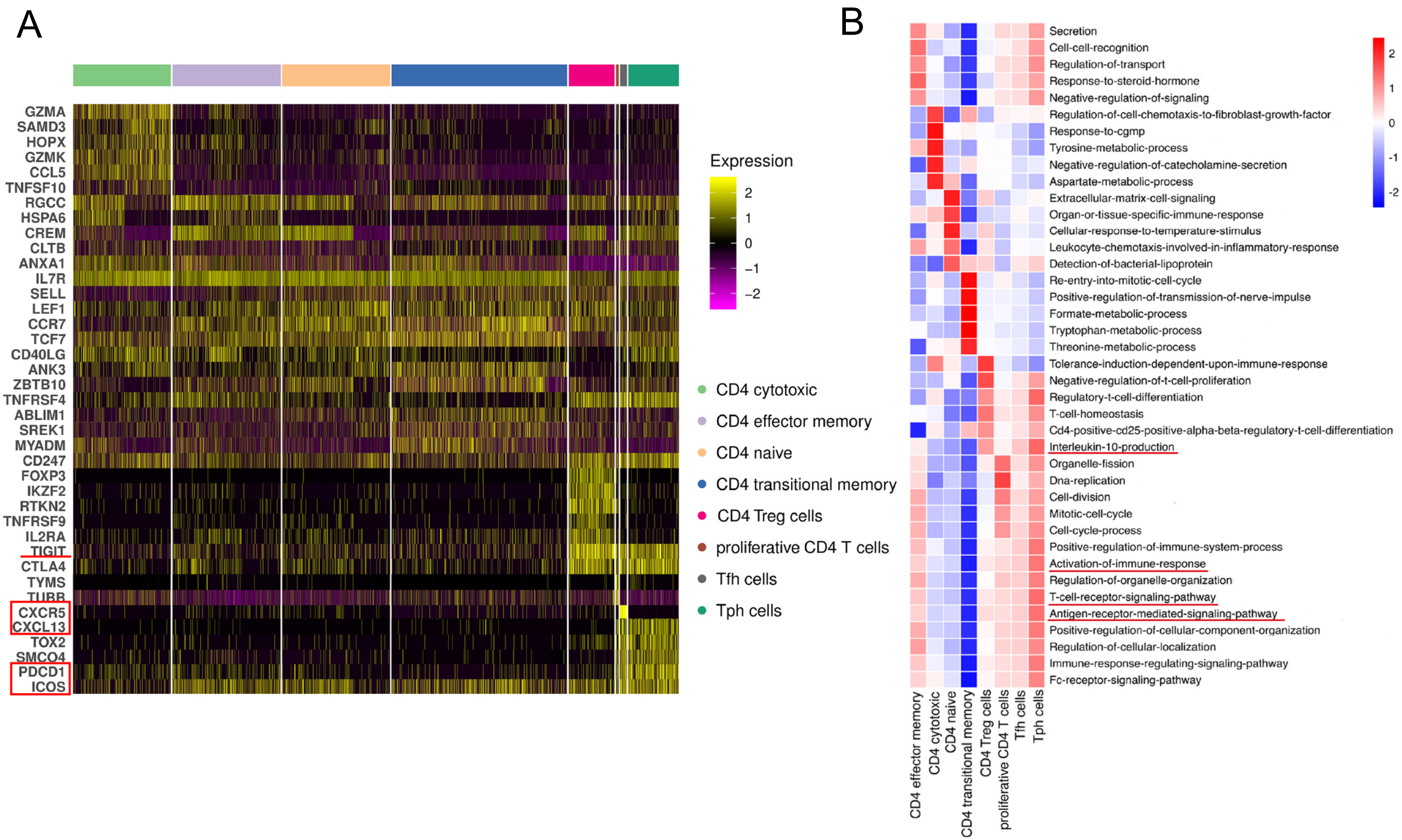

Supplementary Figure S3. (A) Marker genes of different CD4^{+} T cell subtypes. **(B)** GSVA pathway analysis of CD4^{+} T cell subtypes.

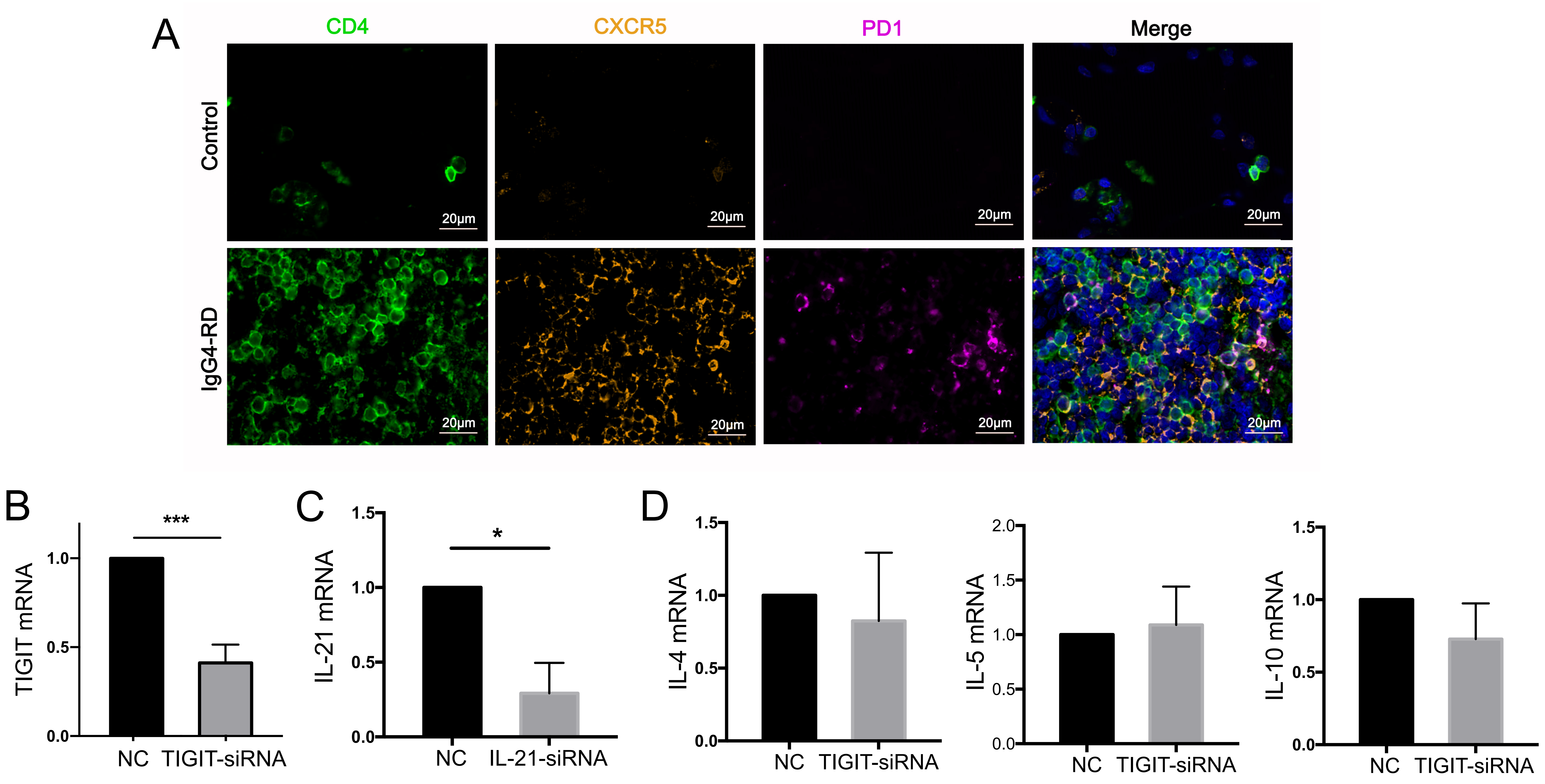

Supplementary Figure S4. **(A)** Tph cells were increased in retroperitoneum tissue of IgG4-RD patients. **(B)** The expression of TIGIT was significantly decreased in $CD4^+$ T cells with TIGIT knockdown. **(C)** The expression of IL-21 was significantly decreased in $CD4^+$ T cells with IL-21 knockdown. **(D)** The expression of IL-4, IL-5, and IL-10 in $CD4^+$ T cells had no significant changes after TIGIT knockdown. $^{*}p < 0.05$, $^{***}p < 0.001$.

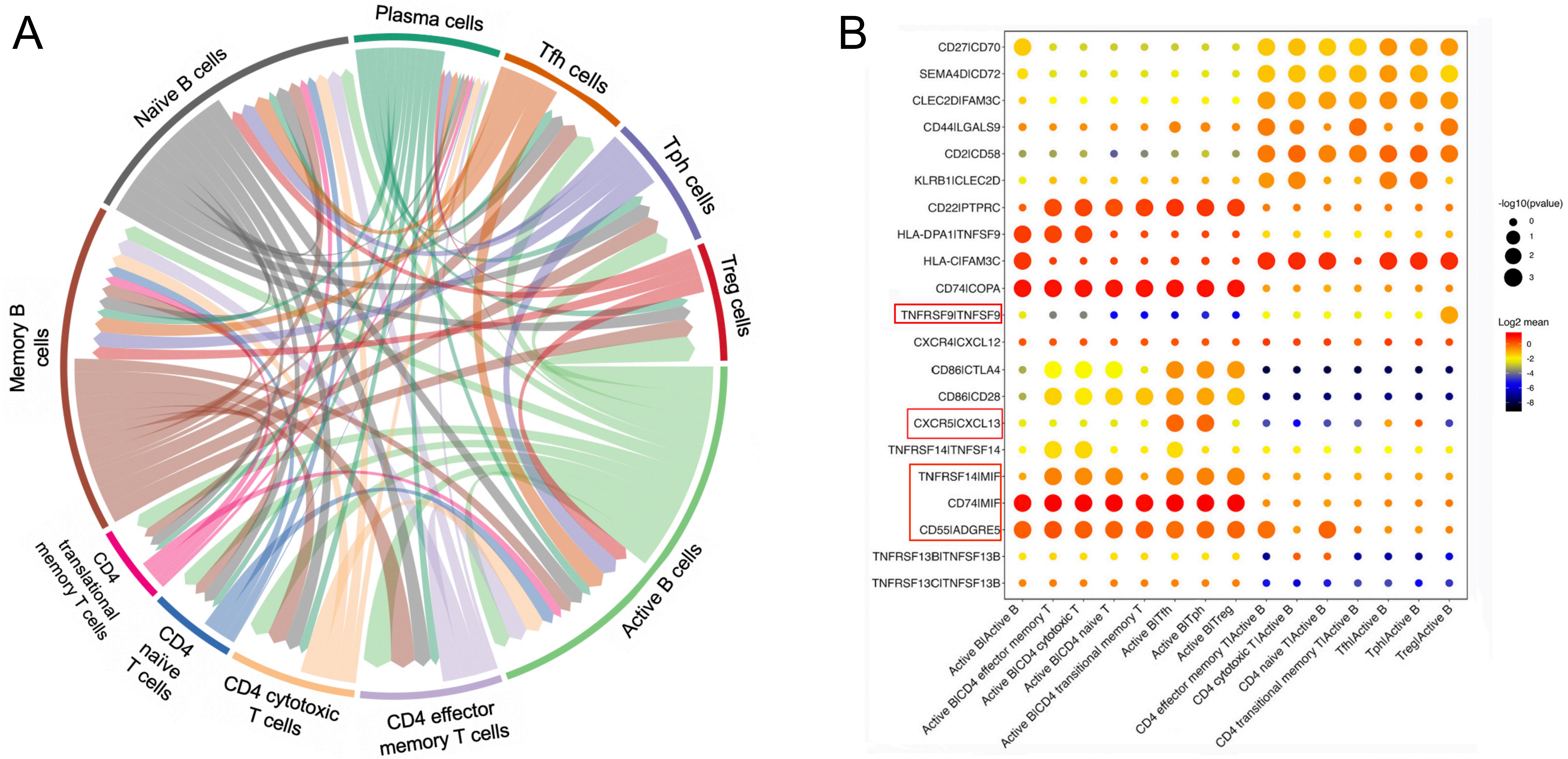

Supplementary Figure S5. (A) Interaction between B cell subtypes and CD4$^+$ T cell subtypes in the IgG4-RPF group. **(B)** Receptor-ligand pairs between active B cells and CD4$^+$ T cells in the IgG4-RPF group

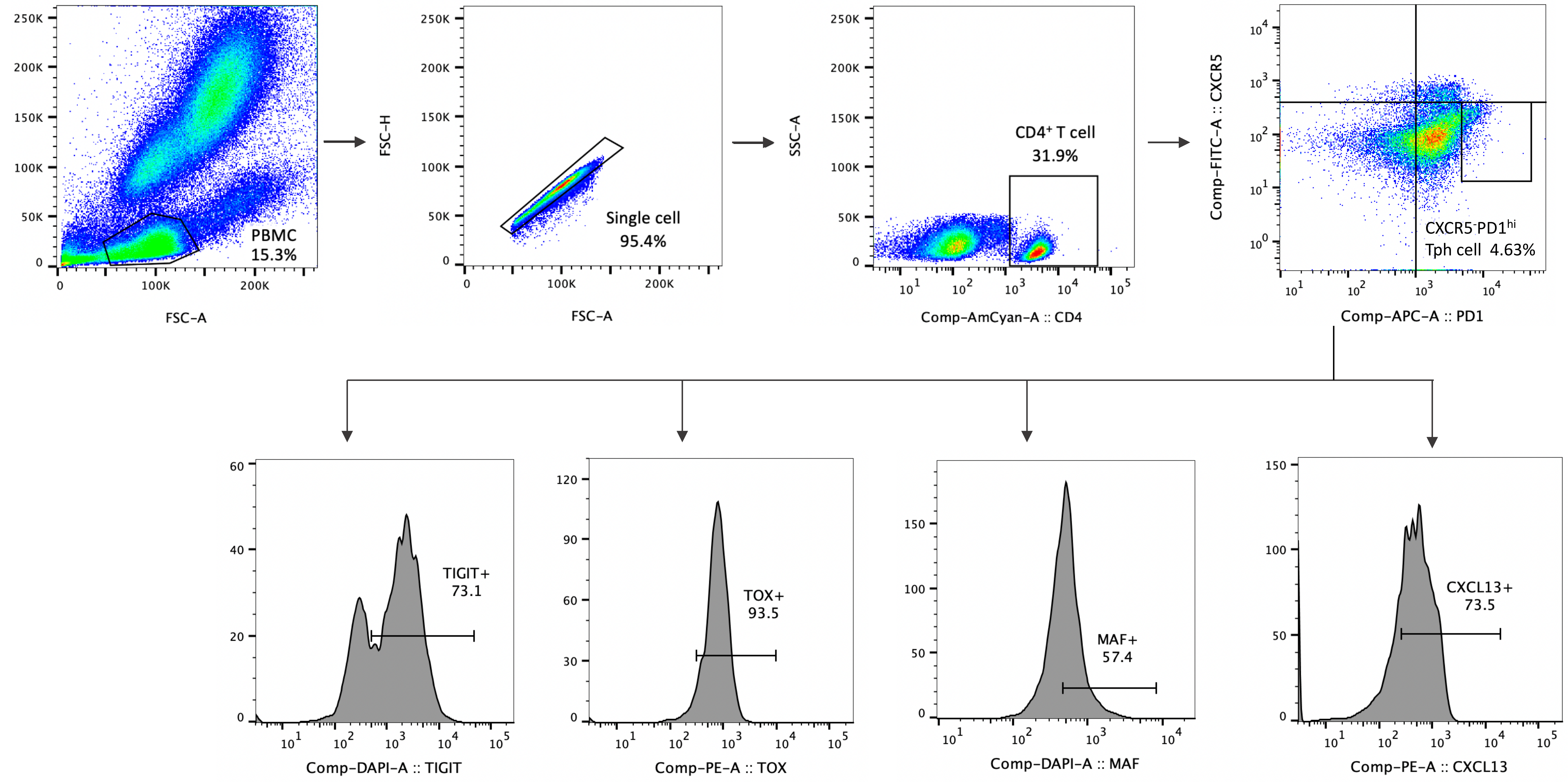

Supplementary Figure S6. The gating strategies of flow cytometry in our study. First, the lymphocytes were gated and doublets were excluded. Then the $CD4^+$ T cells were gated by CD4. Among the $CD4^+$ T cells, $CXCR5^-PD1^{hi}$ Tph cells were gated by PD1 and CXCR5. Subsequently, the expression of TIGIT, TOX, MAF, and CXCL13 in Tph cells were quantified by histogram.
