# Supplementary material for: Single-Cell RNA-Sequencing Reveals Peripheral T Helper Cells Promoting the Development of IgG4-Related Disease by Enhancing B Cell Activation and Differentiation
Source: Int J Mol Sci. 2023 Sep 6;24(18):13735. doi: 10.3390/ijms241813735 (PMC10530310; doi:10.3390/ijms241813735)
Supplement: Supplementary file 1 [file ijms-24-13735-s001.zip › supplementary Methods.pdf]

ScRNA-seq

Quality Control

To remove low quality cells and likely multiplet captures, we filtered out cells with UMI/gene numbers out of the limit of mean value +/- 2-fold standard deviations assuming a Guassian distribution of the UMI/gene numbers for each cell. Following visual inspection of the distribution of cells according to the fraction of mitochondrial genes expressed, we further discarded low-quality cells where >10% of the counts belonged to mitochondrial genes. Additionally, we applied the DoubletFinder package [65] (version 2.0.2) to identify potential doublets. After applying these quality control criteria, the remaining single cells were included in downstream analyses. Library size normalization was performed with the NormalizeData function in Seurat [64] to obtain the normalized count. Specifically, the global-scaling normalization method "LogNormalize" was used to normalize the gene expression measurements for each cell by the total expression, multiplied by a scaling factor (10,000 by default), and the results were log transformed.

Gene analysis, clustering, and identification of the single cells

The top variable genes across single cells were identified using the method described by Macosko et al [66] and the most variable genes were selected using the FindVariableGenes function in Seurat [64]. Principal component analysis (PCA) was performed to reduce the dimensionality with the RunPCA function in Seurat [64]. Graph-based clustering was performed to cluster cells according to their gene expression profile using the FindClusters function in Seurat [64]. Cells were visualized using a 2-dimensional t-distributed stochastic neighbor embedding (t-SNE) algorithm with the RunTSNE function in Seurat. The FindAllMarkers function (test.use = bimod) in Seurat was used to identify marker genes of each cluster. For a given cluster, FindAllMarkers identified positive markers compared with all other cells. Multimodal intersection analysis (MIA) [67] was then used with the reference transcriptomic datasets [36, 68-70] to infer the origin of each of the single cells independently and identify cell types. Differentially expressed genes (DEGs) were identified using the FindMarkers function (test.use = MAST) in Seurat [64]. P-values < 0.05 and $|\log_2\text{fold change}| > 0.58$ was set as the threshold for significantly differential expression. GO enrichment and KEGG pathway enrichment analysis of DEGs were performed using R based on the hypergeometric distribution.

Cell-cell communication analysis

Cell-cell communication was analyzed using CellPhoneDB to identify biologically relevant ligand-receptor (LR) interactions [72]. We defined a ligand or a receptor as "expressed" in a particular cell type if 10% of the cells of that type had non-zero read counts for the ligand/receptor-encoding gene. Statistical significance was then assessed by randomly shuffling the cluster labels of all cells and repeating the above steps, which generated a null distribution for each LR pair in each pairwise comparison between two cell types. After running 1,000 permutations, P-values were calculated with the normal distribution curve generated from the permuted LR pair interaction scores. To define cell-cell communication networks, we linked any two cell types where the ligand was expressed in the former cell type and the receptor in the latter. R packages Igraph and Circlize were used to display the cell-cell communication networks.

Gene set variation analysis (GSVA)

For gene set variation analysis, the GSEABase package (version 1.44.0) [73] was used to load the gene set file which was downloaded and processed from the KEGG database (https://www.kegg.jp/). To assign pathway activity estimates to individual cells, we applied GSVA[4] using standard settings (version 1.30.0). The differences in pathway activities scored per cell were calculated with the LIMMA package (version 3.38.3).

Real-time quantitative PCR

The primers used in this study included:

TIGIT: forward: TGGTCGCGT- TGACTAGAAAGA; reverse: GGGCTCCATTCCTCCTGTC;
IL-4: forward: CGGCAACTTTGTCCACGGA; reverse: TCTGTTACGGTCAACTCGGTG;
IL-5: forward: TCTACTCATCGAACTCTGCTGA; reverse: CCCTTGCACAGTTTGACTCTC;
IL-10: forward: TCAAGGCGCATGTGAACTCC; reverse: GATGTCAAACTCACTCATGGCT;
IL-21: forward: TGTGCAAAGTCAGGATTATTTCCC; reverse AGCCTTCTCCTTCAACCAAGA;
β-actin: forward: CTGGGACGACATGGAG- AAAA; reverse: AAGGAAGGCTGGAAGAGTGC.
