# Supplementary material for: Single-Cell RNA-Sequencing Reveals Peripheral T Helper Cells Promoting the Development of IgG4-Related Disease by Enhancing B Cell Activation and Differentiation
Source: Int J Mol Sci. 2023 Sep 6;24(18):13735. doi: 10.3390/ijms241813735 (PMC10530310; doi:10.3390/ijms241813735)
Supplement: Supplementary file 1 [file ijms-24-13735-s001.zip › supplementary tables.pdf]

Table S1. The clinical features of the 37 patients for peripheral blood validation and tissue validation

Peripheral blood validation					
	**Total IgG4-RD patients (n=24)**	**Patients with active IgG4-RD (n=13)**	**Patients with inactive IgG4-RD (n=11)**	**Healthy Control (n=13)**	***P***
**Age (median, IQR, year)**	56.5 (47-63.75)	56 (49-66)	57 (47-62.5)	47 (45-50)	0.09
**Sex (male, n, %)**	14 (58.3)	8 (61.5)	6 (54.5)	5 (38.5)	0.484
**Single-organ involvement （n, %）**	8.3 (2)	7.7 (1)	9.1 (1)	NA	
**2-organ involvement (n, %)**	22 (91.7)	12 (92.3)	10 (90.9)	NA	
**Serum IgG4 (median, IQR, g/L)**	3.1 (1.41-4.6)	3.8 (3. 5-16.4)	1.5 (1.2-2.4)	NA	
**IgG4-RD RI (median, IQR)**	8 (4-9)	9 (8.75-15)	4 (2.3-4.8)	NA	

Tissue validation				
	**IgG4-RD group (n=10)**	**Patients with benign retroperitoneum tumor (n=5)**	**Patients with Sjögren's syndrome (n=5)**	***P***
**Age (year)**	57.5 (47.8-69.5)	48 (48-57)	42 (36-58)	0.316
**Sex (male, %)**	90 (9)	40 (2)	0 (0)	0.016

IQR, interquartile range; RI, responder index

Table S2. The cell marker of each cell type.

	**Marker**
**B cell**	CD19, CD79A
**Plasma cell**	SLAMF7, IGKC, MZB1
**T cell**	CD3G, CD3D, CD3E
**NK cell**	KLRC1, KLRD1
**Macrophage**	CD68, CD163
**Monocyte**	CD14, CD300E
**Neutrophil**	CXCR1, CMTM2
**pDC**	CLEC4C
**cDC**	FLT3, CD1E
**Fibroblast**	COL1A2, COL1A1, DCN
**Endothelial cell**	VWF, CDH5
**Mast cell**	TPSB2, CPA3, MS4A2
**Smooth muscle cell**	ACTA2, PDGFRA

NK, nature killer; pDC, Plasmacytoid dendritic cell; cDC, classical dendritic cell

Table S3. The proportions of B, plasma, and T cells in the total cells of each sample.

Sample (total cell number)	B cell	plasma cell	T cell
control1 (n=10175)	6.47%	0.93%	25.80%
control2 (n=9328)	0.55%	0.04%	16.34%
control3 (n=8860)	17.12%	1.43%	47.66%
IgG4-RPF1 (n=8017)	23.54%	1.53%	68.75%
IgG4-RPF2 (n=7181)	4.34%	3.15%	54.98%
IgG4-RPF3 (n=7009)	29.36%	0.20%	66.93%

Table S4. The proportions of $CD4^+$ T, $CD8^+$ T, and NK cells in T/NK cells of each sample.

Sample (T/NK cell number)	$CD4^+$ T cell	$CD8^+$ T cell	NK cell
control 1 (n=2625)	47.7%	30.8%	21.5%
control 2 (n=1524)	26.6%	50.7%	22.8%
control 3 (n=4223)	61.9%	23.8%	14.3%
IgG4-RPF 1 (n=5112)	64.7%	25.9%	9.3%
IgG4-RPF 2 (n=3948)	46.6%	24.1%	29.4%
IgG4-RPF 3 (n=4691)	80.2%	14.3%	5.6%
